# What determines a leaf's shape?

**DOI:** 10.1186/2041-9139-5-47

**Published:** 2014-12-22

**Authors:** Jeremy Dkhar, Ashwani Pareek

**Affiliations:** Stress Physiology and Molecular Biology Laboratory, School of Life Sciences, Jawaharlal Nehru University, New Delhi, 110067 India

**Keywords:** Leaf shape, Auxin, Polarity specification, Environmental factors, *Nepenthes*, Morphological novelty

## Abstract

The independent origin and evolution of leaves as small, simple microphylls or larger, more complex megaphylls in plants has shaped and influenced the natural composition of the environment. Significant contributions have come from megaphyllous leaves, characterized usually as flat, thin lamina entrenched with photosynthetic organelles and stomata, which serve as the basis of primary productivity. During the course of evolution, the megaphylls have attained complexity not only in size or venation patterns but also in shape. This has fascinated scientists worldwide, and research has progressed tremendously in understanding the concept of leaf shape determination. Here, we review these studies and discuss the various factors that contributed towards shaping the leaf; initiated as a small bulge on the periphery of the shoot apical meristem (SAM) followed by asymmetric outgrowth, expansion and maturation until final shape is achieved. We found that the underlying factors governing these processes are inherently genetic: *PIN1* and *KNOX1* are indicators of leaf initiation, *HD-ZIPIII*, *KANADI*, and *YABBY* specify leaf outgrowth while *ANGUSTIFOLIA3* and *GROWTH-REGULATING FACTOR5* control leaf expansion and maturation; besides, recent research has identified new players such as *APUM23*, known to specify leaf polarity. In addition to genetic control, environmental factors also play an important role during the final adjustment of leaf shape. This immense amount of information available will serve as the basis for studying and understanding innovative leaf morphologies *viz.* the pitchers of the carnivorous plant *Nepenthes* which have evolved to provide additional support to the plant survival in its nutrient-deficient habitat. In hindsight, formation of the pitcher tube in *Nepenthes* might involve the recruitment of similar genetic mechanisms that occur during sympetaly in *Petunia*.

## Introduction

**Figure 1:**
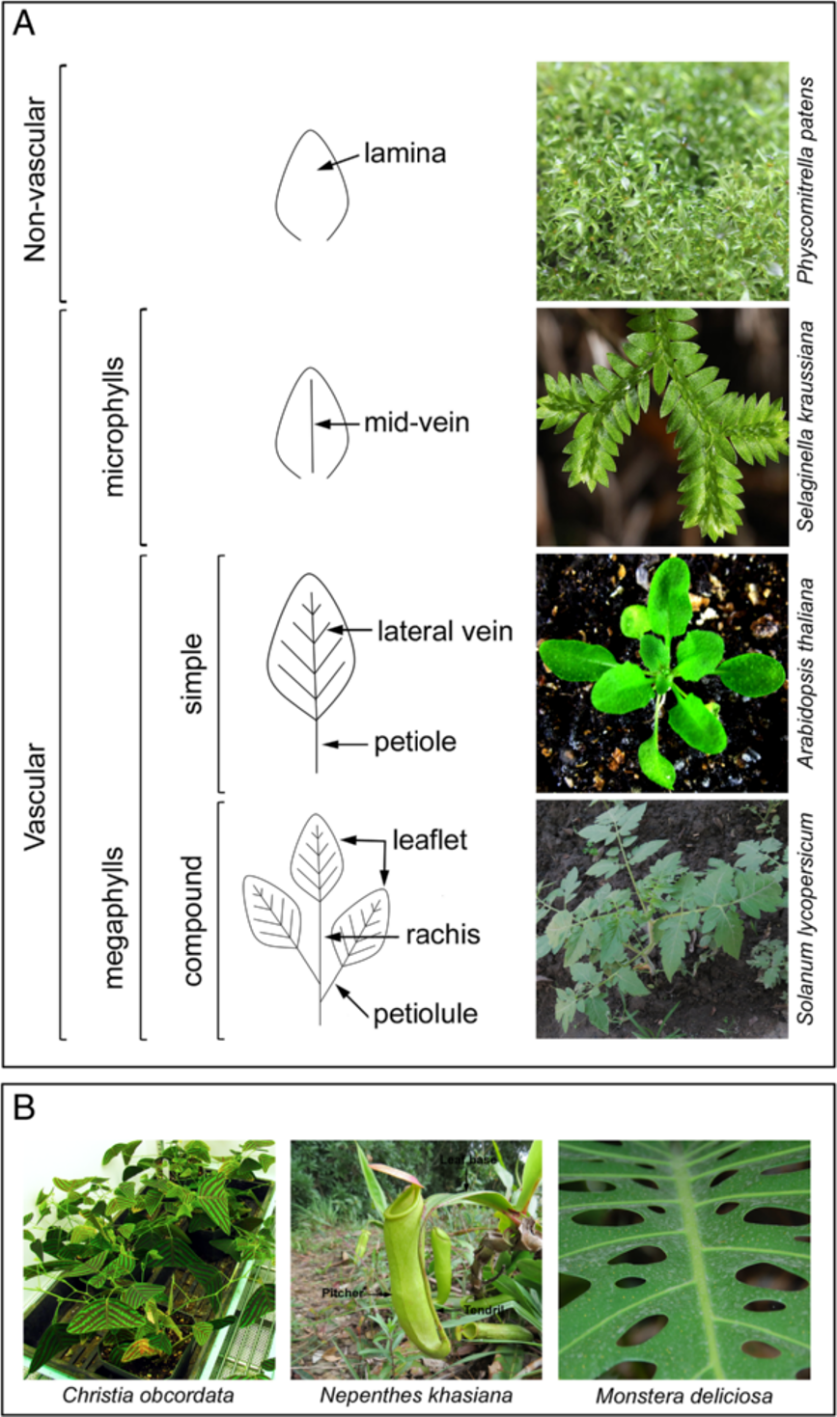
**Diversity in leaf forms across land plants. (A)** Selected representatives of the different types of leaf forms found in non-vascular and vascular model plant species *viz. Physcomitrella patens* (non-vascular), *Selaginella kraussiana* (microphyll), *Arabidopsis thaliana* (simple megaphyll), and *Solanum lycopersicum* (compound megaphyll). **(B)** Selected representatives of uncommon and innovative leaf morphology found in vascular non-model plant species *viz. Christia obcordata* (butterfly-shaped leaf), *Nepenthes khasiana*, and *Monstera deliciosa* (modified leaf). Contributors of photographs used in the figure can be found in the Acknowledgements section.

In comparison to the vibrant colors of the flower, the ‘leaf’ has nothing special to offer as most are green-colored attributed to the presence of chlorophyll. But their attractiveness lies in their varying shapes and sizes; from the uncommon butterfly-shaped leaf of *Christia obcordata* to the extensively studied ovate-shaped leaf of *Arabidopsis thaliana* (Figure 1A and B). This variation, arising due to several factors, offers great functional significance that influences plant success [1]. In the case of leaf size, the explanation has been straightforward; it decreases with increasing altitude, decreasing rainfall, and soil nutrient content [2, 3]. Moreover, smaller-sized leaves are better adapted to hot or dry environments [4]. However, in case of leaf shape, environmental influences *viz.* light, temperature, and so on have been difficult to explain [2]. Nonetheless, these factors and most importantly light, play special roles in the final adjustment of leaf shape [5]. But the tremendous variations observed in leaves are mostly attributed to their genetic control - the control of gene regulatory networks (GRNs) and signaling pathways that make a leaf, from a small bulge on the SAM, into a fully developed lateral outgrowth with diverse shapes. Although poorly understood, herbivory is another factor contributing to leaf shape variation [6]. Due to continued interest in this area of research, a review on the factors that determine a leaf its shape is called for. And though a similar review is available in the literature, this [5] was published almost a decade ago. Therefore, a revisit on the topic is warranted and we intend to comprehensively cover all aspects of leaf shape development that span across vascular plants with a focus on angiosperms. Our aim is to summarize these development events and the underlying mechanisms that govern them, and highlights recent advances culminating with a discussion on directions for future research. In fact, the present review lay more emphasis on the genetic control with a brief overview on the environmental components. This remarkable information garnered may open up avenues for a probable shift from model to non-model plant species showing morphological novelties, for example, pitchers of the carnivorous plant *Nepenthes*, modified from an otherwise unexceptional leaf (leaf base) through the formation of tendrils (Figure 1B). A note on this interesting plant genus with unusual leaf form is also presented and discussed at the concluding section.

## Review

### Definition, origin, and evolution of a leaf

From a layman’s perspective, ‘leaf’ is a flattened, green-colored structure laterally attached to the stem. This perception may be too simple considering the remarkable diversity that leaves exhibit. As a prerequisite to their review on angiosperm leaf shape, Nicotra *et al.* [1] defined a leaf as a ‘vascular asymmetric appendicular structure initiated at the shoot apical meristem’. This definition is applicable to all vascular plants, but does not hold true for bryophytes (mosses, liverworts, and hornworts) as they lack a well-defined system of vascular tissue [1]. In fact, the leaf-like structures of bryophytes share no homology with leaves of vascular plants. But for an organ to be considered a leaf, other basic, but delicate, morphological connections that include the dorsiventrality of the leaf and distinctive meristem distribution in relation to their symmetrical arrangement on the axis may be taken into consideration [7]. Dorsiventrality or the distinctness of the upper and lower surfaces of the leaf is evident in all land plants; prominent in vascular plants but to a lesser extent in bryophytes, observed mainly in the midrib region referred to as ‘costa’. Besides this attribute of dorsiventrality, leaves become determinate, planar, and laminar structures. Considering all these views, we described a leaf as a determinate laminar structure with distinct adaxial and abaxial surfaces, formed, developed, and arranged in a particular manner on the flanks of an indeterminate SAM.

Vascularization, however, is an important anatomical characteristic that defines the two leaf types observed in vascular plants: microphylls (single vasculature) and megaphylls (complex vasculature, Figure 1A). But vasculature is not the only distinguishing feature; size (small or large) and leaf gaps (absence or presence) also differentiate the two leaf types with complexity more pronounced in megaphyllous leaves [8–11]. Examples of microphylls and megaphylls are evident in lycophytes (extant clubmosses, spikemosses, and quillworts) and euphyllophytes (comprising the extant ferns, horsetails, and seed plants), respectively. These contrasting morphological characteristics displayed correlates with an independent origin and evolution of the microphyllous and megaphyllous leaves. The two leaf types are believed to have evolved independently from simple leafless vascular plants around 480 and 360 million years ago [12]. The microphyllous leaf emerged during Late Silurian/Early Devonian era while the megaphyllous leaf evolved during the late Devonian period [12, 13]; the latter event is linked with a 90% drop in atmospheric CO_2_ that corresponds with a 100-fold increase in stomatal density to avoid lethal overheating [14]. Three hypotheses have been proposed for the origin of the microphylls, but Bower’s [15] ‘Enation theory’ is the most plausible as it is supported by an evolutionary series of related Devonian taxa, *Sawdonia* (and *Discalis*), *Asteroxylon*, and *Drepanophycus* ([16] and references therein). For megaphyll evolution, Zimmermann’s telome theory has been widely accepted as the leading explanation which involves ‘overtopping, planation and webbing’ - three fundamental steps that transform a telome into a laminated leaf blade [17]. This theory of megaphyll evolution is similar in concept to ‘evolutionary tinkering’, a phenomenon that involve changes in already existing organs/forms. Furthermore, the evolution of the megaphylls occurred at least twice, once in ferns and horsetails and the other in seed plants that include the gymnosperms and angiosperms [18]. Among angiosperms, dissected or compound leaf form found in *Cardamine hirsuta,* pea, tomato, and so on have evolved independently from simple leaves [19].

Interestingly, the independent evolution of microphylls and megaphylls does not correspond with unique mechanisms of leaf formation; rather, common developmental mechanisms could underlie microphyll and megaphyll formation [20]. This proposition was also corroborated by a recent finding that suggests a common GRN for protonema and root hair development in *Physcomitrella patens* (bryophyte) and *Arabidopsis thaliana*, respectively [21]. Earlier, mutational and gene silencing work on four distantly related species *viz. Aquilegia caerulea*, *Solanum lycopersicum* and *S. tuberosum*, *Cardamine hirsuta*, and *Pisum sativum*, showed that a common underlying mechanism involving NAM/CUC3 genes promoted compound leaf development [22]. Therefore, the remarkable diversity in leaf form is a result of the common regulatory networks recruited and remodeled during the course of land plant evolution. We begin our review with the genetic basis of leaf shape determination.

### Genetic basis of leaf shape: genetic interactions, gene expression patterns, microRNAs, and active hormonal regulations

Attainment of the final sizes and shapes of the plant leaf involves three major developmental events: begins with leaf initiation, followed by leaf outgrowth, and ends with leaf expansion and maturation. We highlight below the underlying genetic mechanisms that control these events. As a complement to the text, a list of all the participating genes, their functions, and mutant phenotypes is summarized in Table 1.

**Table 1:** **Genes involved in major developmental events of the leaf**

Developmental events	Genes	Description	Biological function	Mutant phenotype	Plant species	References
Leaf initiation	*PIN-FORMED1 (PIN1)*	Transmembrane protein	Auxin efflux	Develop naked, pin-shaped inflorescences; leaves become fused; phyllotaxy disrupted	*Arabidopsis thaliana*	[23–25]
	Class-1 *KNOTTED*-like homeobox (*KNOX1*)	Homeodomain protein	Maintain stem cell identity	Loss-of-function mutants failed to develop SAM; gain-of-function mutants showed ectopic SAMs on leaves	*Arabidopsis thaliana*; *Zea mays*	[26, 27]
	*WUSCHEL (WUS)*	Homeodomain protein	Maintain shoot and floral central meristem identity	Delayed growth; disorganized rosette leaves; inflorescence meristem defective	*Arabidopsis thaliana*	[28]
	*CLAVATA (CLV)*	*CLV1* (receptor kinase); *CLV2* (transmembrane protein); *CLV3* (extracellular protein)	Maintain stem cell size	Enlarged shoot and floral meristems; stem overgrowth; additional floral organs	*Arabidopsis thaliana*	[29, 30]
	*ASYMMETRIC LEAVES1/ ROUGH SHEATH2/ PHANTASTICA (ARP)*	MYB domain protein	Stem cell differentiation	Stunted growth; polarity defects; unlike *as1* and *rs2*, *phan* leaves are radialized	*Arabidopsis thaliana*; *Zea mays*; *Antirrhinum majus*	[31–34]
Leaf outgrowth: proximodistal patterning	*KNOTTED1 (KN1)*	Homeodomain protein	Maintain stem cell identity	Gain-of-function mutants displayed flaps of sheath tissue at leaf blade margin; leaf bifurcated	*Zea mays*	[35]
	*LIGULELESS NARROW-REFERENCE (LGN-R)*	Serine/threonine kinase	Establishment of blade/sheath boundary	Heterozygotes displayed narrower and shorter leaves; homozygotes failed reproductive development	*Zea mays*	[36]
Leaf outgrowth: dorsoventral patterning	*PHANTASTICA (PHAN)*	MYB domain protein	Stem cell differentiation	Loss-of-function *phan* mutants develop needle-like leaves lacking dorsoventrality	*Antirrhinum majus*	[37]
	*ASYMMETRIC LEAVES2 (AS2)*	LOB domain protein	Leaf venation pattern and lamina development	Develop narrow and curly leaves with alteration in adaxial/abaxial polarity	*Arabidopsis thaliana*	[38]
	class III *HOMEODOMAIN-LEUCINE ZIPPER (HD-ZIPIII)*	Homeodomain and leucine zipper domain protein	Leaf polarity, meristem function	Lateral organs radialized with adaxial cell fate transformation; modification in vascular patterning	*Arabidopsis thaliana*	[39, 40]
	*KANADI (KAN)*	GARP domain protein	Leaf polarity specification	Develop narrow adaxialized lateral organs; ectopic outgrowths on leaves; gain-of-function mutants displayed abaxialized cell types; blade expansion inhibited	*Arabidopsis thaliana*	[41, 42]
	*APUM23*	PUF RNA-binding protein	Leaf polarity specification	Radialized leaves; disorganized vascular pattern	*Arabidopsis thaliana*	[43]
	*AUXIN RESPONSE FACTOR* (*ARF*)	Protein with N-terminal DNA binding domain, activator/repressor domain, C-terminal dimerization domain	Leaf polarity specification	Narrow leaves with ectopic blade outgrowths	*Arabidopsis thaliana*	[44]
	*miR165*	21-nucleotide non-coding RNAs	Leaf polarity specification, meristem function, vascular development	Loss of SAM; altered organ polarity; defective vascular development	*Arabidopsis thaliana*	[45]
	*miR166*	21-nucleotide non-coding RNAs	Leaf polarity specification, meristem function, vascular development	Enlarged SAM; enhanced vascular development	*Arabidopsis thaliana*	[46]
	*RNA-DEPENDENT RNA POLYMERASE6 (RDR6)/DICER-LIKE4 (DCL4)*	RNA-dependent RNA polymerase/RNase-III enzyme	Leaf polarity specification	Accelerated juvenile-to-adult phase transition; early development of adult lateral organs; lack ta-siRNAs	*Arabidopsis thaliana*	[47–49]
Leaf outgrowth: mediolateral patterning	*YABBY (YAB)*	Protein with zinc-finger and helix-loop-helix domains	Leaf polarity specification, lamina expansion	Minuscule and bushy plants with loss of lamina expansion and polarity defects	*Arabidopsis thaliana*	[50, 51]
	*Narrow sheath (ns)*	Homeodomain protein	Leaf founder cell recruitment, leaf expansion	Develop extremely narrow leaves; short internode	*Zea mays*	[52, 53]
	*PRESSED FLOWER (PRS)*	Homeodomain protein	Marginal cell proliferation	Smaller sepals; defective marginal regions	*Arabidopsis thaliana*	[54]
	*MAEWEST (MAW)*	Homeodomain protein	Organ fusion and lateral expansion	Severe leaf blade reduction, thickened leaf margins; petal expansion reduced; defective carpel fusion	*Petunia × hybrida*	[55]
	*YUCCA (YUC)*	Flavin monooxygenase	Leaf and vascular development, floral patterning	Stunted growth with curved leaves; smaller inflorescence meristem; defective floral and leaf vasculature	*Arabidopsis thaliana*	[56, 57]
Leaf expansion and maturation	*ANGUSTIFOLIA* (*AN3*)/*GRF-INTERACTING FACTOR1(GIF1)*	Transcription coactivator	Cell expansion	Reduced leaf width and length; petal width reduction; more leaf number	*Arabidopsis thaliana*	[58, 59]
	*GROWTH-REGULATING FACTOR5 (GRF5*)	Transcription activators containing N-terminal QLQ or WRC domain	Cell proliferation	Loss-of-function mutants displayed narrow leaves and petals; gain-of-function mutants develop	*Arabidopsis thaliana*	[58, 59]
	*CINCINNATA (CIN)*	TCP domain protein	Cell proliferation	Develop large crinkly leaves	*Antirrhinum majus*	[60]
Leaf margin alterations	*miR164A*	Non-coding miRNA	Leaf margin development	Enhanced leaf margin serration in loss-of-function mutants; gain-of-function mutants develop leaves with smooth margins	*Arabidopsis thaliana*	[61]
	*CUP-SHAPED COTYLEDON2* (*CUC2*)	Protein containing the NAC DNA-binding domain	Shoot meristem formation; organ boundary specification; leaf margin development	Produced leaves with smooth margins	*Arabidopsis thaliana*	[61]
	*PIN-FORMED1 (PIN1)*	Transmembrane protein	Auxin efflux	Loss-of-function mutants develop smooth leaf margins	*Arabidopsis thaliana*	[62]
	*DEVELOPMENT-RELATED PcG TARGET IN THE APEX* (*DPA*)	RAV transcription repressor	Organ initiation and development; leaf margin development	Loss-of-function mutants showed increased leaf margin serrations and enlarged petals; gain-of-function mutants possessed smooth margins	*Arabidopsis thaliana*	[63]

### Leaf initiation: *KNOX*repression and auxin accumulation

**Figure 2:**
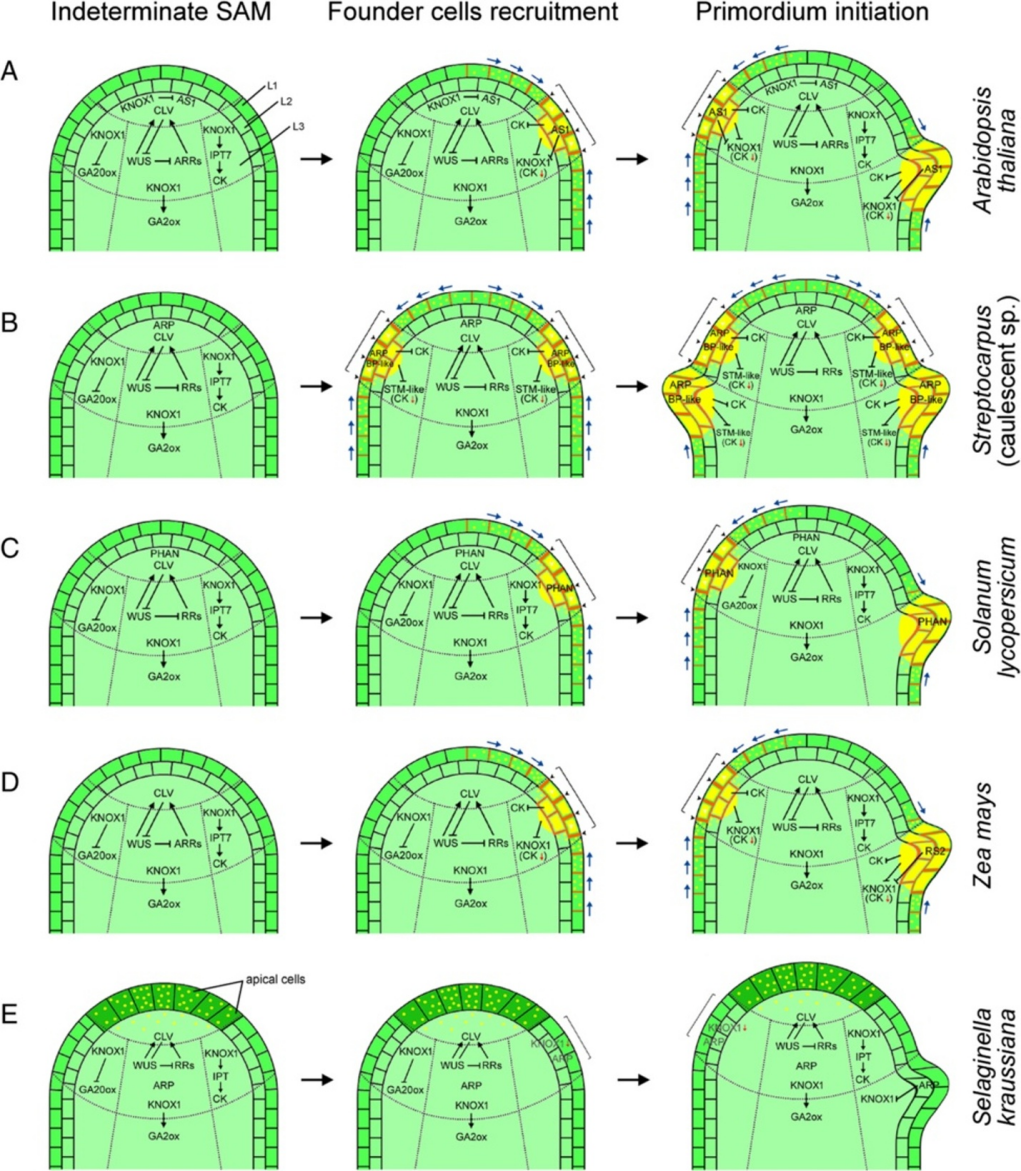
**Diagram illustrating stages of leaf initiation in selected model plant species** ***viz.*** **(A)** *Arabidopsis thaliana*; **(B)** caulescent *Streptocarpus* sp. (simple leaf eudicots); **(C)** *Solanum lycopersicum* (compound leaf eudicot); **(D)** *Zea mays* (simple leaf monocot); and **(E)** *Selaginella kraussiana* (microphyll). Black arrowhead indicates PIN1 polarization; white arrowhead denotes auxin maxima; blue arrow shows the direction of auxin flow; black arrow represents upregulation; blunt end indicates repression; red arrow depicts downregulation; yellow dots represent auxin; square bracket indicates leaf founder cells recruitment sites. Illustrations are adapted from Byrne *et al.* [31] for *A. thaliana*; Nishii *et al.* [84] for *Streptocarpus* sp. (caulescent); Koltai and Bird [85] for *S. lycopersicum*; Timmermans *et al.* [33] and Tsiantis *et al.* [32] for *Z. mays*; Harrison *et al.* [20] and Sanders and Langdale [83] for *S. kraussiana*. L1, L2 = tunica; L3 = corpus.

Studies on model plant species have revolutionized our understanding on the early events of leaf initiation (Figure 2A-E). The findings showed that leaf initiation begins with the recruitment of founder cells, approximately 100 in numbers for *Nicotiana tobacum* [64] and *Gossypium barbadense* [65], at the flanks of the SAM. In eudicots, subpopulations of cells are recruited for leaf initiation while in monocots recruitment of founder cells can occur all along the circumference of the SAM [66, 67]. The initiation of microphyll primordia in *S. kraussiana* also occurs at the periphery of the SAM [68]; however, fewer founder cells (12 to 16) are recruited [69]. But what drives leaf initiation at the periphery of the SAM is yet to be fully ascertained, despite the fact that it corresponds with the concurrent repression of class-1 *KNOTTED*-like homeobox (*KNOX*) genes [26, 70, 71] and local auxin accumulation mediated predominantly by the auxin efflux carrier PIN-FORMED1 (PIN1) [23–25] (Figure 2A-D). In *Arabidopsis thaliana* (henceforth referred to as Arabidopsis), the transmembrane protein PIN1 is strongly expressed in epidermal cells of the SAM and its apical polarization results in the creation of an auxin gradient with maxima directed towards sites of incipient leaf primordia [23–25, 72–75] (Figure 2A). This in turn acts as an auxin sink that is transported basally to promote formation of provascular tissues, creating a field of auxin depletion around the incipient and bulging primordia [76, 77]. The change in auxin transport corresponds with strong PIN1 expression in the central vasculature of developing leaf primordia [25]. In other plant species, for example, maize, the expression pattern of PIN1 is identical to Arabidopsis, although ZmPIN1 localization is also observed in the corpus (L3) of SAM [78]. In *C. hirsuta*, PIN1 facilitate leaflet formation through high auxin activity in the margin of the leaf rachis [79]. These observations suggest that the distribution of auxin maxima, either in the meristem flank or leaf margin, determine where leaf/leaflet primordia originate. Besides auxin concentration and flow, the control of PIN1 localization and expression has also been attributed to mechanical stresses, occurring due to tight interactions between growing cells [80]. Recently, a membrane-bound protein BIG is thought to affect PIN1 protein level by regulating its transcription [81]. However, the exclusive role of PIN1 in leaf initiation is still debatable owing to the normal development of *pin1* mutant leaves during early vegetative growth in Arabidopsis [23, 24]. In an attempt to understand this surprising development, Guenot *et al.* [82] investigated other plasma-membrane localized PIN proteins (PIN2, PIN3, PIN4, and PIN7) to uncover if they could compensate for the loss of PIN1 during rosette leaves formation in Arabidopsis. Surprisingly, none of these proteins were expressed in the SAM, suggesting that other auxin transporters, auxin synthesis, and auxin-independent mechanisms of leaf initiation in Arabidopsis exist [82]. Recent evidences from *S. kraussiana* suggest that the underlying molecular mechanisms for polar auxin transport (PAT) are likely to be conserved across all vascular plants [83]; however, auxin does not promote leaf initiation in *S. kraussiana* [83]. This finding implies that an auxin-independent mechanism for leaf initiation in *S. kraussiana* exists that remained conserved throughout vascular plants evolution and recruited during early vegetative growth in Arabidopsis.

Another critical event occurring at the SAM prior to the initiation of leaf primordia is the downregulation of *KNOX1* genes (Figure 2A-E). KNOX1 proteins function in the maintenance of stem cell identity - mutational studies have shown that Arabidopsis and maize plants with loss-of-function mutations in *KNOX1* genes failed to maintain SAM [26, 27] - and repression of these genes changes the indeterminacy state of stem cells to determinate ones. This organogenic switch is controlled by the relative amount of two phytohormones: cytokinin (CK) and gibberellin (GA), responsible for cell division and cell elongation, respectively [86]. High CK to low GA ratio promotes indeterminacy of SAM while low CK to high GA ratio facilitates determinacy. In Arabidopsis, the high CK to low GA ratio is achieved through KNOX1 proteins via upregulation of cytokinin biosynthesis genes *isopentenyl transferase* 7 (*IPT7*) [87, 88] and repression of GA20-oxidase gene [89]. In maize, GA level is reduced by a direct upregulation of the GA catabolism gene *ga2ox1* [90] (Figure 2D). Similarly, *GA2ox2* mRNA level in Arabidopsis leaves increased in response to higher levels of cytokinin and *KNOX1* expression [87]. Another *KNOX*-independent genetic pathway involving WUSCHEL (WUS) and CLAVATA (CLV), which acts in the central zone of meristem, control stem cell fate by direct regulation of cytokinin-inducible response regulators [28–30, 91] (Figure 2A-E).

The low CK to high GA ratio is attained through different pathways of *KNOX1* downregulation. One of the pathways is mediated by auxin through polar transport at sites of incipient leaf primordia thereby repressing *KNOX1* [92] and CK signaling [93, 94] (Figure 2A-D). Another pathway involves MYB transcription factors encoded by the ARP genes. ASYMMETRIC LEAVES1 (AS1) of Arabidopsis [31], rough sheath2 (rs2) of maize [32, 33], and PHANTASTICA (PHAN) of Antirrhinum [34] (hence the name ARP) are explicitly expressed in lateral organs founder cells and negatively regulate respective *KNOX1* gene expression (Figure 2A, D). This requires the interaction of AS1 and RS2 with HIRA, a chromatin-remodeling factor that could alter local chromatin organization at the *KNOX1* loci [95]. It becomes evident that the ARP/KNOX regulatory module is mutually exclusive, common in most simple leaved species with the exception of *Streptocarpus*, wherein KNOX1 and ARP are co-expressed in leaf primordia [84, 96] (Figure 2B). Co-expression of ARP/KNOX module is also observed in most compound leaved species and their expression pattern varies from one species to another [85, 97] (Figure 2C). Interestingly, the ARP/KNOX module in *Selaginella* is either mutually exclusive (leaves and stem) or overlapping (meristem) [20] (Figure 2E). This co-expression might facilitate shoot bifurcation in *Selaginella* [20], delays maturation of the compound leaf to allow leaflet formation [98], and promotes macrocotyledon growth and meristem development in *Streptocarpus* [84]. Recent evidence has suggested another mode of KNOX repression involving an epigenetic interaction between Arabidopsis AS1-ASYMMETRIC LEAVES 2 (AS2) complex and POLYCOMB REPRESSIVE COMPLEX (PRC) 2 to stably silence the stem cell regulators [99]. The *AS2* gene encodes an AS2/LOB domain-containing protein comprising a cysteine repeat motif and a leucine-zipper-like sequence in its amino-terminal half [100]. Hence, the multiple levels of regulation may explain the crucial role that *KNOX1* genes play in leaf development because mis-expressions have resulted in adverse phenotypes [101], thereby reducing plant success.

### Leaf outgrowth: change in division pattern along three axes

**Figure 3:**
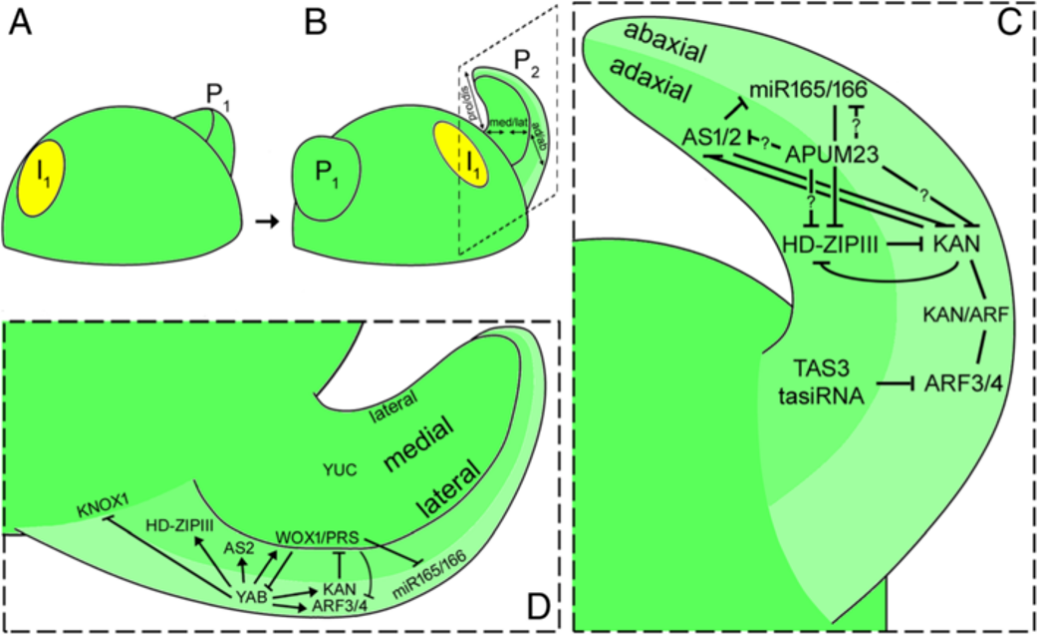
**Diagram illustrating leaf outgrowth in Arabidopsis. (A)** Leaf primordium initiation; **(B)** leaf outgrowth; **(C)** adaxial/abaxial patterning (magnified view of inlet in B depicts the underlying genetic mechanisms controlling adaxial/abaxial patterning); **(D)** medial/lateral patterning (magnified view of inlet in B shows the underlying genetic mechanisms controlling mediolateral patterning). Illustrations are adapted from references mentioned in the text. P_1_: plastochron 1; P_2_: plastochron 2; I_1_: incipient site showing auxin maxima (yellow circle). Pro/dis: proximal/distal; med/lat: medial/lateral; ad/ab: adaxial/abaxial.

At the phenotypic level, leaf initiation is recognized by the appearance of a bulge at sites on the periphery of the SAM where *KNOX1* repression and auxin maxima occur (Figure 3A). Immediately after primordial initiation, determinate cells are induced to change division pattern along three axes: proximal/distal, adaxial/abaxial, and medial/lateral (Figure 3B). Each axis is discussed below.

### Proximal/distal patterning: so much yet so little known

The proximal/distal axis is established with the initiation of leaf primordia [35, 102]. Its determination was thought to be independent of dorsalizing function, as needle-like leaves of *phan* mutants having no lateral outgrowth retained their proximodistal axis [37]. However, evidences from mutants of KANADI and YABBY gene family (regulators of adaxial/abaxial polarity) displaying shorter leaf length have suggested otherwise [103]. Ramirez *et al.* [35] showed that gain-of-function *knotted1* (*kn1-DL*) mutants produced flaps of sheath-like tissue along the maize leaf margins caused by the misexpression of *kn1* in these regions. This finding highlights the probable role of KN1 in proximal/distal polarity, creating a juxtaposition of proximal (*kn1* expressing) and distal cells (blade) in *kn1-DL* mutants [35]. Recently, Moon *et al.* [36] identified a new mutation in maize, *Liguleless narrow-Reference* (*Lgn-R*), mapped to a grass-specific kinase. Homozygous *Lgn-R* mutants displayed reduced leaf width and length, are significantly shorter in height and lack reproductive organs as compared to wild type, suggestive of its role in proximal/distal patterning [36]. Although no definite genetic marker(s) has been found associated with proximodistal axis, these studies have paved the way towards identifying genes specifying proximal/distal axis in leaf development.

### Adaxial/abaxial patterning: class III *HD-ZIPs*, microRNAs, *KANADI*, and auxin interacting factors

Experiments to establish the mechanism of adaxial/abaxial polarity in leaf development started around 60 years ago through surgical incisions separating incipient primordia from the apical meristem [104, 105]. Resulting potato leaves were mostly centric and abaxialized caused either by cessation of apical growth or elimination of its effect [104]. Laser-based techniques to ablate tomato leaf primordia yielded similar results, producing plants (65%) with partial or complete loss of lateral leaflets and removal of the L1 layer of the SAM eliminated dorsoventral polarity [106]. These results suggest that a signal emanating from the SAM is required for normal adaxial/abaxial patterning and draw attention to the L1 layer playing a role in the establishment of the adaxial domain of leaf primordia. Similar phenotypes were observed in loss-of-function *phan* mutants of *Antirrhinum majus*, identifying *PHAN* as a determinant for maintaining the adaxial identity [37]. They demonstrated a relationship between adaxial/abaxial polarity and lamina outgrowth, and hypothesized that the juxtaposition of adaxial and abaxial identity promotes lamina outgrowth. Although *PHAN* and its orthologues (referred as *ARP* gene family) are uniformly expressed in young leaf primordia of respective species, their roles in adaxial specification are not strictly conserved. Abaxialization due to knockdown of *PHAN* orthologues is observed in certain lineages viz. *LePHAN* in tomato [107], but not in maize (*RS2*) [33] or Arabidopsis (*AS1*) [31]. Surprisingly, overexpression of AS2 in Arabidopsis resulted in plants with narrower curly leaves displaying dramatic alteration in the identity of both adaxial and abaxial epidermal cells and the abaxial side showed mostly adaxial features [38]. Furthermore, mutants of the *indeterminate gametophyte1* (*ig1*) gene in maize, sharing sequence similarity with *AS2* of Arabidopsis, produced leaves with defective adaxial/abaxial specification [108]. These studies highlight the role of AS2 in maintaining adaxial identity.

The adaxial/abaxial axis is also established at leaf initiation, and represents an important axis that require proper establishment for proper lamina outgrowth. In Arabidopsis, *PHABULOSA* (*PHB*), *PHAVOLUTA* (*PHV*), and *REVOLUTA* (*REV*), members of the class III *HOMEODOMAIN-LEUCINE ZIPPER* (*HD-ZIPIII*) gene family, play vital roles in adaxial/abaxial polarity specification, and are expressed in the adaxial domain of developing leaf primordia to specify adaxial cell fate [39, 109] (Figure 3C). Arabidopsis plants with dominant mutations in *phb* and *phv* developed rod-shaped or trumpet-shaped leaves with adaxial characters around their circumference [39] while gain-of-function alleles of *rev* resulted in adaxialized lateral organs mediated by microRNAs - miR165 and miR166 [40]. In fact, expression of HD-ZIPIII genes in the abaxial domain are repressed by miR165/166 [45, 46]. Ectopic/constitutive overexpression of *MiR165* and *miR166* produced contrasting phenotypes with comparable reduction in transcript levels of *HD-ZIP III* genes [45, 46]. In rice, four out of five class III *HD-ZIP* genes, *OSHB1* to *OSHB4*, control adaxial/abaxial patterning and are similar in gene structure and expression patterns to Arabidopsis *HD-ZIPIII* genes. Mutations in the miR166-binding sites of these *OSHB* genes, particularly *OSHB1* and *OSHB3*, resulted in leaf polarity defects with varying degree of severity [110]. These studies suggested a conserved functional role of *HD-ZIPIII* genes in Arabidopsis, rice, maize (*rolled leaf1* (*rld1*) and *leafbladeless1* (*lbl1*) [111]) and most likely across angiosperms [112].

Abaxial identity in leaves requires the function of *KANADI* gene family, encoding nuclear-localized GARP-domain transcription factors, which are expressed in the abaxial domain of leaf primordia (Figure 3C). In Arabidopsis - of which four *KAN* (1-4) genes are present - loss-of-function *kan1* mutants showed apparent disruption in adaxial/abaxial cell gradient as compared to wild type whereas transgenic seedlings, with *KAN1* fused to a constitutive CAMV 35S promoter, developed elongated and pointed cotyledons with no subsequent leaves production [41]. Similar results were obtained when *KAN1*, *KAN2*, and *KAN3* genes were ectopically expressed using the 35S promoter, but severe alteration in leaf polarity occurred in *kan1 kan2* double mutants [42]. *kan1 kan2* plants develop narrow cotyledons and leaves with ectopic outgrowths on their abaxial side, and displayed adaxialized lateral organs, particularly petals and carpels [42]. Eshed *et al.* [103] extended their study on the triple mutants of *KAN* (1-3) and observed that mutant leaves, although radialized initially, maintained some level of polarity during development. Interestingly, the expression pattern of the *PHB* gene in the *kan1 kan2 kan3* background was altered, expressing throughout the developing leaf with a maximum level at the adaxial domain indicating that *KAN* genes antagonistically regulate *HD-ZIPIII* genes [103] (Figure 3C). A recent study corroborated this finding, indicating that *KAN* and *HD-ZIPIII* have opposing effects on genes that are involved in auxin biosynthesis and transport, for example, TAA1, NPH3-like genes, and so on, while *PIN4* is repressed by *KAN* [113]. This finding highlights the importance of the adaxial/abaxial pathway in patterning auxin synthesis, transport, and signaling. Since this antagonistic effect arises during cotyledon formation; it is assumed that similar responses could occur during leaf development [113]. *APUM23*, encoding PUF RNA-binding protein family, has been identified as a new player of leaf polarity specification in Arabidopsis [43] (Figure 3C). Mutation in *APUM23* increases the severity of *kan1 kan2* mutant leaf phenotypes, displaying enhanced reduction in blade expansion. Moreover, RT-PCR revealed overexpression of the two *KAN* genes as well as *PHB*, *REV*, *AS1*, and *AS2* in the triple mutant as compared to wild type [43]. These evidences suggest that APUM23 act to indirectly regulate the expression of major adaxial/abaxial leaf polarity genes [43].

The other gene family known to specify abaxial cell fate is the *AUXIN RESPONSE FACTOR* (*ARF*) family, which function by binding to auxin response elements on promoters of auxin response genes and transduce auxin signal during plant growth and development. Evidence that points to their role in adaxial/abaxial polarity emerged from mutational studies and expression pattern of *ETT* (also known as *ARF3*) and *ARF4* in Arabidopsis [44]. Severe polarity defects were observed in *ett-1 arf4-1* and *ett-1 arf4-2* double mutant plants resulting in abaxialized leaves similar to *kan1 kan2* mutants [44], suggesting a key relationship between *KAN* and *ARF*. Evidently, a direct interaction between ETT and KANADI (KAN1 and KAN4) was reported, and their overlapping expression pattern suggests common regulatory function in polarity establishment and organogenesis [114] (Figure 3C). In turn, both *ETT* and *ARF4* were earlier shown as targets of *TAS3* derived *trans*-acting siRNAs (ta-siRNAs) and displayed marked upregulated expression in siRNA- and miRNA-defective mutants [115] (Figure 3C). In Arabidopsis, loss-of-function mutations in key *TAS3* ta-siRNA biogenesis genes, encoding RNA-DEPENDENT RNA POLYMERASE6 (RDR6) and DICER-LIKE4 (DCL4), resulted in plants with accelerated juvenile-to-adult phase transition and early development of adult lateral organs characteristics as compared to wild type [47, 48]. But when a ta-siRNA-insensitive *ETT*, generated by introducing silent mutations into target sites, or *ETT* were expressed in a *rdr6-15* mutant background, defects in leaf morphology was observed [49]. The transgenic plants displayed narrow, highly twisted, curly and irregularly shaped leaves or in severe cases, the appearance of deeply lobed leaves with ectopic radial leaf primordia on the abaxial surface [49]. These results specify *TAS3* ta-siRNAs as negative regulators of abaxial cell fate through the regulation of *ETT* and *ARF4* [49], thereby identifying small RNAs as key players of adaxial/abaxial polarity specification.

### Medial/lateral patterning: role of *YABBY*and *WOX*gene family

Proper establishment and juxtaposition of the adaxial and abaxial domain is required for lamina outgrowth, which initiates at the adaxial/abaxial boundary and develop along an axis referred to as the medial/lateral axis (Figure 3B and D). As lamina outgrowth involves cell division followed by cell elongation and differentiation, the adaxial/abaxial boundary formed in early developing leaf primordia represents another leaf meristematic zone called plate meristem or blastozone [116, 117]. The morphogenetic capacity of the blastozone ensures formation of the lamina and other structures such as lobes and leaflets; improper or loss of lamina outgrowth is a consequence of defective leaf adaxial/abaxial polarity as evident from mutant analyses mentioned above. Although initially thought to be a major component of abaxial cell fate specification because of its expression pattern and gain-of-function alleles [118], the *YABBY* gene family is a primary player of medial/lateral specification (Figure 3D). In Arabidopsis, six members of the *YABBY* gene family have been identified [119] and are known to encode transcription factors with a zinc-finger and a helix-loop-helix motif. These include *FILAMENTOUS FLOWER* (*FIL*), *YABBY2* (*YAB2*), *YAB3*, and *YAB5*, which are expressed in leaf primordia, and *CRABS CLAW* (*CRC*) and *INNER NO OUTER* (*INO*) that are localized to the floral organs. Double mutants of *fil* and *yab3* genes displayed partially radialized leaves but maintained adaxial/abaxial polarity to a larger extent as both adaxial and abaxial surface cell types can be easily distinguished [50]. Sarojam *et al.* [51] extended their investigation to all four vegetative *YABBY* genes and found that the severity of polarity defects and loss of lamina outgrowth were more pronounced in triple mutant *fil-8 yab3-2 yab5-1* (*yab135*) and quadruple mutant *fil-8 yab2-1 yab3-2 yab5-1* (*yab1235*) plants as compared to the double mutants, though initial polarity establishment remained intact. These results indicated that the lack of lamina outgrowth and polarity maintenance is associated with the loss of YABBY function. The *YABBY* genes are regulated by players involved in adaxial/abaxial polarity specification *viz. KANADI*, *HD-ZIPIII*, *AS* [103, 111, 120] (Figure 3D). This was also verified by a recent study that identified KAN1 and ARF4 as positively regulated targets of FIL/YAB3 and vice versa [121] (Figure 3D).

Analyses on genes of the *WUSCHEL* (*WUS*)*-RELATED HOMEOBOX* (*WOX*) family shed further light on the mechanism of lateral organ outgrowth through evidences that emerged from preliminary studies on *narrow sheath* (*ns1*) and *ns2*, two duplicated genes of the *WOX* family found in maize. The *ns* mutant plants displayed extremely narrow leaves, but their length were not compromised as compared to wild type plants [52], suggesting an unconnected relationship between the medial/lateral and proximodistal axes. *In situ* hybridization revealed that the expression of the *ns1* and *ns2* genes in maize occurs in the lateral domains of shoot meristem and the margins of young lateral organ primordia [53], thereby hinting at their involvement in promoting lamina outgrowth. In Arabidopsis, localization of the lateral-axis dependent gene *PRESSED FLOWER* (*PRS*) specified similar pattern of expression as the *ns* genes, spatiotemporally expressed in the margins of sepals, petal, stamens, and developing leaves [54]. Furthermore, gain-of-function mutants of the *PRS* gene resulted in epidermal outgrowths on sepal margins while loss-of-function mutant displayed defects in the marginal regions of sepal, indicating that *PRS* is essential for the proliferation of marginal cells [54]. Isolation and characterization of *MAEWEST* (*MAW*) gene, which encodes a member of WOX family, in *Petunia* × *hybrida* highlighted its role in lamina outgrowth specification [55]. Double mutants of *MAW* and *CHORIPETALA SUZANNE* (*CHSU*) resulted in severely defective lamina outgrowth displaying mostly abaxialized cell types at the leaf margins. *WOX1*, an Arabidopsis *MAW* ortholog, mutants showed no apparent abnormal phenotype, but when crossed with *prs* mutants, resulting F2 populations displayed narrow leaf lamina and thickened leaf margins similar in phenotype to *maw* mutants, indicative of their redundant roles in promoting lateral lamina outgrowth [55, 112]. *WOX1* is expressed in the leaf meristem (plate meristem), overlapping with *PRS* [54, 122]. Based on the expression levels of *PRS* and *WOX1* genes in *YABBY* gene family mutants (*fil yab3* and *fil yab3 yab5*) and *kan1 kan2* mutants backgrounds, *WOX1* was shown to be upregulated by *YABBY* genes while *PRS* remained unaffected and KAN represses both genes in the abaxial domain of leaf primordia [123] (Figure 3D).

During leaf morphogenesis, free auxin is systematically reallocated from the tip of the leaf (site of initial synthesis) to the expanding leaf blade margins, finally ending in the midvein of the lamina [124]. This pattern of free auxin synthesis facilitates leaf blade outgrowth. Several lines of evidence support this notion, for example, broad exogenous application of IAA across one side of the developing leaf primordium of *Solanum lycopersicum* resulted in ectopic lamina outgrowth with maintained compoundness and asymmetry [125]. Similarly, at sites where ectopic auxin accumulation appeared - indicated through PIN1 expressions - as in the hypocotyls of *kan1 kan2 kan4* triple mutants, ectopic leaf-like organs developed [126]. Furthermore, formation of ectopic bulges at the sides of developing leaf primordium of *yabby* quadruple mutants corresponds to sites where secondary PIN1 convergence points occurred [51]. More convincing results emerged from studies of *YUCCA* (*YUC*) gene family encoding flavin monooxygenase-like enzymes involved in local auxin biosynthesis. Mutations in four (*yuc1246*) of the 11 Arabidopsis *YUC* genes caused severe defects on plant stature and leaf development (narrow leaves), besides other developmental processes such as vascular and floral patterning [56, 57]. When mutated *YUC124* genes were constructed in *as2 rev* and *kan1 kan2* backgrounds (polarity defective mutants), severely defective phenotypes with extremely narrow leaves was observed [127]. Interestingly, the pentuple mutants lack the finger-shaped protrusions evident in *as2 rev* and *kan1 kan2* mutants formed as a result of ectopic juxtaposition of the leaf adaxial and abaxial domains [103, 128]. Wang *et al.* [127] showed that these protrusions represent hydathodes-like structures, thereby suggesting that *yuc* genes, in response to adaxial-abaxial juxtaposition, promote leaf margin development and blade outgrowth via local auxin accumulation [112].

### Leaf expansion and maturation

Once the establishment of leaf polarity along the three-dimensional axes is achieved, leaf begins to expand until it acquires its final size and shape. Prior to cell expansion, cells divide and grow, that is, proliferate. Proliferation occurs early during leaf development and spreads throughout the leaf primordia [129, 130]. At this stage, cells undergo successive mitotic cell cycle exemplified by expression pattern of *CYC1* [129] and the presence of cells with variable C-DNA content [131]. Genes that are exclusively expressed at this phase includes members of A- and B-type cyclin family, known to control the activity of cyclin-dependent kinases (CDKs) and other downstream transcriptional complexes [132]. Once cell proliferation ceases, cells immediately switch to expansion mode via endoreduplication. It may be mentioned here that certain species such as *Aquilegia vulgaris*, *Lactuca serriola*, and *Oryza sativa* show little or no endoreduplication event, despite their small genome size [131]. Initiation of endoreduplication is indicated by the emergence of cells with 8C and 16C DNA content [132]. At this stage, cell cycling pattern partition the leaf into three regions: the proliferative cells containing basal region, the distal region that comprises expanding cells and the boundary that separates the basal/distal region termed the cell cycle arrest front. The progression of the cell cycle arrest front during the transition phase is an abrupt process [133, 134], and the timing of its appearance is an important factor for determining the final size of the lateral organ [130]. When expansion terminates, cells become mature. Maturation is indicated by the increasing levels of KRP proteins that inhibit cell cycle progression, and stable DNA distribution and cell number [132].

Previous genetic analyses have identified key regulatory components of cell proliferation. Interaction between the transcription coactivator *ANGUSTIFOLIA3* (*AN3*) and transcription factor *GROWTH-REGULATING FACTOR5* (*GRF5*) has been shown to regulate leaf size and shape; mutation in these genes resulted in plants with narrow-leaf and decreased cell number [58, 59]. More recent evidence has emerged that linked cell proliferation and adaxial/abaxial patterning as well. Mutational studies of the *AN3* and *AS2* genes showed that *an3* enhances leaf polarity defects in *as1* and *as2* mutants [135]. But the narrow-leaf phenotype of *an3* is a consequence of a growth defect rather than a polarity defect, which implies that AN3 act at a specific developmental phase to regulate cell proliferation and polarity specification [135]. Besides *GRFs*, *CYTOKININ RESPONSE FACTOR2* (*CRF2*), *CONSTANS-LIKE5* (*COL5*), *HECATE1* (*HEC1*), and *ARABIDOPSIS RESPONSE REGULATOR4* (*ARR4*) were identified as prominent transcription factors that are regulated by AN3 through binding to SWITCH/SUCROSE NONFERMENTING (SWI/SNF) chromatin remodeling complexes [136]. Other regulators of cell proliferation include *CINCINNATA* (*CIN*), a member of the TCP gene family which contains the bHLH motif that permits DNA binding and protein-protein interactions [137]. *CIN* mutants of *A. majus* displayed enhanced cell proliferation at the leaf margins producing large crinkled leaves [60]. Overexpression of *TCP4*, a *CIN*-like TCP gene, disrupted normal leaf morphogenesis resulting in small cup-shaped leaves due to early onset of maturation and decreased cell proliferation [138].

In an attempt to understand how leaf size and shape varies among plants, Kuchen *et al.* [139] devised an experimentally validated model to help define the evolution and development of diverse organ shapes. The model correctly matches the observed growth dynamics and shape changes of leaf 1 in Arabidopsis. To account for leaf shape other than leaf 1, the authors varied the effects of two factors, among the many specified: PGRAD, defined to express as a linear gradient along the proximodistal axis, and LAM, defined to express everywhere. Varying the effects of PGRAD at the distal end and the strength of promotion by LAM resulted in the generation of diverse morphospace resembling some of the botanically described leaf shapes (for example, obcordata, ovate, and elliptic). The underlying genes that may explain these patterns include *LEAFY PETIOLE* and *YABBY* genes as candidates of LAM factor while *CUC* genes may underlie PGRAD factor [139]. This model, which also accurately predicts the growth patterns of Antirrhinum leaves [139], provides a framework for the experimental testing of the control of organ shape in diverse plant species.

### Leaf margin alterations: *mir164A*, *CUC2*, PIN1, and *DPA4*are key players

**Figure 4:**
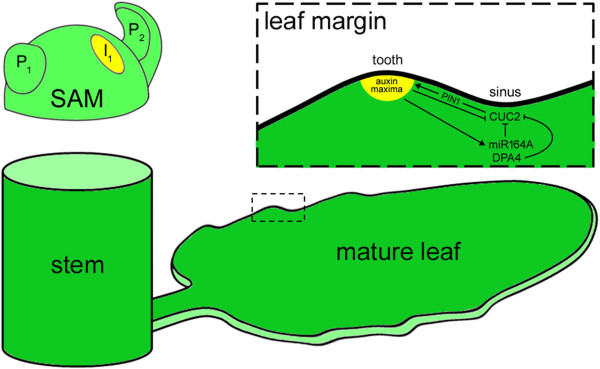
**Diagram illustrating leaf margin development in Arabidopsis.** Magnified view of inlet shows the underlying genetic mechanisms controlling this process. Illustrations are adapted from Nikovics *et al.* [61], Bilsborough *et al.* [141], and Engelhorn *et al.* [63]. P_1_: plastochron 1; P_2_: plastochron 2; I_1_: incipient site showing auxin maxima (yellow circle).

Growth and development in all three axes transforms the small bulging leaf initials on the periphery of the SAM into a flattened structure of varying sizes and shape. If leaf development were to stop here, we might expect leaf margins of the same type. But the characteristic nature of the leaf margin and the underlying mechanisms that exist confers additional complexity resulting in leaves of diverse marginal leaf shapes. Leaf margins are of different types: entire, serrate or lobed; it was until 2006 that the molecular mechanisms of leaf margin serration in Arabidopsis could be elucidated. In Arabidopsis, serration in leaves become more pronounced as the plant develop, with early rosette leaves showing less serration as compared to the ones that developed later ([61] and references therein), and this has been shown to be controlled by *mir164A*, *CUC2*, PIN1, and *DPA4* [61, 63, 140, 141] (Figure 4). Knock-out mutations in *mir164a* resulted in plants with deeper serrations as compared to wild type plants, caused as a result of a disruption in the *miR164*-dependent regulation of *CUC2*, a member of the *NAC* gene family [61]. In *mir164a cuc2* double mutant plants, leaf serration is lost suggesting that *CUC2* play a key role in the development of serrated leaf margins in Arabidopsis, and the degree of serration depends on the balance between the co-expressed *MIR164A* and *CUC2* genes [61]. Contrary to the findings of Nikovics *et al.* [140], Kawamura *et al.* [140] showed that *CUC2* promotes teeth outgrowth rather than suppressing the sinus growth.

Serration initiates at sites where auxin maxima occurs, as evidenced by expression of the auxin response sensor *DR5::GFP*, with concurrent repression of *CUC2* [141]. Eliminating this interspersed distribution through exogenous application of auxin and continuous *CUC2* expression at the marginal domain resulted in leaves with smooth margins. Furthermore, based on PIN1 localization in *cuc2* mutants, Bilsborough *et al.* [141] showed that *CUC2* expression is required to induce PIN1 convergence points in the leaf margins. These results show the existence of a PIN1-mediated feedback regulatory loop between *CUC2* and auxin [141] (Figure 4). Loss of *PIN1* function resulted in plants with smooth margins [62]. In another recent study, it was shown that *DEVELOPMENT-RELATED PcG TARGET IN THE APEX* (*DPA*) genes contributed to the late-stage development of leaf margin serration in Arabidopsis [63]. T-DNA inserted *DPA4* lines displayed enhanced leaf margin serrations while *35S::DPA4* overexpressor lines lacked serrated leaf margins. *In situ* hybridization and qRT-PCR analysis indicated that *CUC2* expression in *35S::DPA4* lines were strongly downregulated hinting at an additive role by *DPA4* in repressing *CUC2* expression and thereby confirming the crucial role of *CUC2* in leaf margin serrations formation in Arabidopsis [63] (Figure 4). Besides *CUC2*, *CUC3* was also shown to promote Arabidopsis leaf serration, but acts later in development mainly for teeth growth maintenance [142]. Earlier, ectopic *KNAT1* expression transformed simple Arabidopsis leaves into lobed leaves, and lobing was shown to initiate at sites where leaf margin serration develop [143]. Double transgenic Arabidopsis lines ectopically overexpressing *KNAT1* and *PALMATE-LIKE PENTAFOLIATA1* (*PALM1*), a Cys(2)His(2) zinc finger transcription factor involved in compound leaf development in *M. truncata*, displayed normal leaves suggesting a *PALM1*-mediated repression of *KNAT1* via regulation of downstream targets [144]. Leaf margin serration is apparent in compound-leafed species as well, such as *M. truncata*; here, serration is confined to the distal part of leaflets. As was reported in Arabidopsis [141], leaf distal margin development in *M. truncatula* requires the auxin efflux protein MtPIN10 [145]. Plants with mutated *PIN10* gene exhibited complete loss of serration [145]. In a recent study, insertion mutation in the *MtPHAN* gene resulted in plants with deeper serrations as compared to wild type, suggesting that *MtPHAN* also play key roles in leaf margin development [146]. When both *mtphan mtpin10* genes were mutated, the compound leaves displayed smooth margins [146], confirming the crucial role of PIN10 in distal leaf margin development of *M. truncata*. Genetic evidences from simple and compound leaved-species identify transmembrane PIN proteins as crucial players of leaf margin development.

### Environmental basis of leaf shape: biotic and abiotic components

Our understanding on the genetic basis of leaf shape diversity has come from the enormous amount of research conducted on model plant species. In the process, evidences that point to the role of environmental cues on leaf shape determination emerged. For example, the *phan* mutants of *Antirrhinum majus* analyzed for dorsiventrality specification displayed varying phenotypes when grown at different temperatures [37]. At 17°C, leaves are needle-like, reverting to normal types at higher temperature (25°C), suggesting that PHAN expression respond differently and reveal the sensitivity of other gene components towards temperature changes [37]. This observation suggests that external factors play a role in shaping lateral organs. An overview on some of the environmental factors controlling leaf shape development is presented below.

### Role of temperature and light in leaf shape diversity

Because of their fluctuating tendency, temperature and light regimes could adversely affect leaf growth processes and leaf shape [147]. Royer *et al.* [148] studied the effect of temperature gradient on leaf shape plasticity in *Acer rubrum* grown at two gardens with contrasting climates (Rhode Island and Florida, USA). Plants at Rhode Island garden, with mean annual temperature (MAT) of 9.8°C, displayed highly dissected leaves with more number of teeth as compared to plants grown at Florida garden (MAT = 20.0°C). This observation was corroborated by another study that spans 92 globally distributed and climatically diverse sites, and reported that plants found in colder climates develop larger, higher number of teeth and highly dissected leaves [149]. These results showed the apparent impact of environmental change on leaf shape variations.

But among all the causative environmental factors, which includes elevated CO_2_ [150] and gravity [5], light forms an important physical component that has tremendous impact on leaf shape [5, 67]. Differences in light intensity resulted in plants with varying leaf forms: low intensity induces petiole elongation with reduced blade expansion whereas high intensity promoted blade expansion but inhibits the elongation of the leaf petiole [5]. More convincing results appeared from a study that showed how light affect leaf initiation and positioning [151]. In dark conditions, tomato seedlings ceased to initiate proper leaf development, but reassumed growth when transferred into light conditions [151], a response comparable to pea [152]. Moreover, the dark-grown seedlings displayed slender leaves as compared to light-grown seedlings. Based on the results that emerge through the use of norflurazon, a photosynthesis inhibitor, cessation of leaf initiation in tomato seedlings is independent of photosynthesis. Yoshida *et al.* [151] extended their study on tomato *aurea* mutants that lack proper phytochrome photoreceptor and found retarded leaf formation and irregular phyllotaxy in the mutants. These results suggest that light acts as a morphogenic signal that requires signaling molecule (auxin and cytokinin) to transduce its effect during leaf development.

In naturally limiting light conditions such as the understorey tropical rainforests, fascinating leaf variation exists. Plants of the genus *Monstera* develop holes in adult leaves, referred as leaf fenestration (Figure 1B). The display of leaf fenestration in adult leaves is intriguing and often rare, which lack convincing evolutionary explanation. In an attempt to reveal the basis of this morphological peculiarity, Muir [153] designed a model to test the hypothesis that leaf fenestration might offer adaptive significance for survival in the dark understorey tropical rainforests. Muir [153] used the model to compare between fenestrated (top) and entire (bottom) juvenile leaves where leaf area and mean daily leaf photosynthesis are same in both leaf shapes. Although the fenestrated leaf utilized less sunlight, intercepted as sunflecks (brief, intermittent, and unpredictable periods of direct light), as compared to entire leaves, the average carbon gain worked out is same. However, variance in canopy growth rate is lower in the fenestrated leaf. The model demonstrated that fenestration can reduce the variance in plant growth thereby increasing plant fitness, and this was shown to depend on the stochastic sources of light (sunfleck) for carbon gain [153]. It can be assumed that the tropical rainforest habitat imposed a selective pressure that drives the development of leaf fenestration.

### Leaf shape variations as a response to herbivory

Selection as a means that coerced the evolution of leaf size and shape has unexpected participation from the animal kingdom, particularly vertebrates and insects, through herbivory [6]. It was proposed that some of the variations seen in plants, for example, highly divided and dissected leaves, heteroblasty and interspecific differences in leaf form, may have evolved as a response to herbivory, to reduce the feeding efficiency or recognition by herbivores [6]. The theory was tested on the highly variable rosette leaves of *Capsella bursa-pastoris* for feeding preferences by adult flea beetles, *Spodoptera* caterpillars, and adult vine weevils [154]. Field and laboratory data for flea beetles, showing preferences for deeply lobed leaves, disprove the theory while Spodoptera caterpillars displayed no preference at all. The adult vine weevils, however, preferred undivided over divided leaves [154]. A similar experiment was conducted on *Ipomoea hederacea*, a plant with two genotypes showing either heart-shaped leaves (genotype 1) or both heart-shaped and three-lobed leaves (genotype 2), to study the effect of leaf shape on insect consumption and performance [155]. Interestingly, the heart-shaped genotypes suffered less damage from foraging by *Spodoptera exigua* (beet armyworms) as compared to lobed-shaped genotypes, but showed no significant effect between juvenile and adult heart-shaped leaves [155]. The above results suggest that herbivory as a means towards leaf shape determination lacked convincing and corroborative results, and this may be attributed to several additional factors that could influence the experimental outcome.

## Conclusions

We conclude and recapitulate that leaf development and the diverse forms it attained is governed by complex genetic interactions, changes in gene expression patterns, participation of microRNAs, and active hormonal regulations, some of which are reprocessed during development or the specification of leaf types. Moreover, the effects of environmental factors in shaping lateral organs are also evident and probably act at a later stage of development for final adjustment. This evidence has expanded our knowledge on the mechanism of leaf development and shape determination; however, our understanding is limited to a few model plant species. In spite of tremendous progress in the field, gaps still exist. The findings that auxin does not promote leaf initiation in *S. kraussiana* nor does it affect leaf development in young *pin1* mutants of Arabidopsis indicate the existence of an auxin-independent mechanism. In a remarkable finding and one that downplayed the role of auxin in apical dominance, Mason *et al.* [156] identified sugar as the crucial regulator of axillary bud outgrowth in plants. As a complement to this finding and an indication that sugar may play a role in leaf development, transcription factors that regulate genes involved in sugar signaling were highly expressed in the basal zone of maize leaf, a region where cell division and cell-fate specification occur [157]. Future research in these directions should hold promise in enhancing our knowledge of the initial events of leaf development. Following the recent discovery of APUM23 as a new regulator of leaf polarity specification, questions have arisen concerning their direct targets (among the known leaf polarity genes). But some of the old questions have remained unresolved, for example, what are the markers that specify proximodistal patterning or what is the nature of the SAM-derived signal required for normal adaxial/abaxial patterning? These and many more have eluded clarification. In addition, major breakthroughs in this field have come from research on plants with megaphyllous leaves. While certain studies have indicated conservation among genes involved in the initiation of megaphylls and microphylls (for example, KNOX, ARP) [20], some have suggested distinct functions (for example, role of class III HD-ZIPs in adaxial/abaxial polarity) [68]. To have a better understanding on the concept of leaf development across land plants, more research into microphyll development is indeed required.

Finally, taking into consideration the enormous amount of leaf shape diversity that plants exhibit, a shift into non-model plant species showing morphological novelties may be envisaged. One such example is the carnivorous plant genus *Nepenthes*, a remarkable botanical entity that is of significant interest in the context of plant adaptation. *Nepenthes*, especially *N. khasiana* (Figure 1B), typically grow in nutrient-deficient soil (particularly nitrogen) and in order to survive have developed specialized organs called pitchers, modified through a process of epiascidiation that involves in-rolling of the adaxial leaf surface followed by marginal fusion [158, 159]. These pitchers have the ability to attract and capture insects, digest them, and ultimately absorb the nutrients. We understand why *Nepenthes* develop pitchers, but how it does remains a mystery? But with the advent of new high-throughput sequencing technologies, this mystery may be unfolded. So what valuable insight could a study on leaf development in *Nepenthes* offer? First, it would significantly contribute towards understanding the evolution of plant development, especially those that are adaptive in nature. Second, it would provide additional insights into the evolutionary origins of leaflike structures, and third, help in understanding how evolution works so as to develop strategies that will enable engineering and improvement of crop plants. Furthermore, the notion that *Nepenthes* pitchers are more specialized in carnivory as compared to other carnivorous plants [160] further justify this proposal. The origin of the pitcher is analogous to that of the leaf (particularly the megaphylls); the latter evolved in correlation with a drop in atmospheric CO_2_ [14] and the former is presumably linked with soil N_2_ reduction, although the association has not been proved yet. This phenomenon of carnivory is considered an ‘opportunity to uncover macroevolutionary patterns and processes that may be generalized to other structural phenomena in angiosperms’ [159]. We now know that simple leaves are determinate appendages; whether pitchers represent determinate morphological structures as well or are modifications that occur at later stages of development is a notion to fathom on. It is a known fact that auxin plays an important role in leaf development; how it controls pitcher development is another interesting aspect that can be looked at? Based on the available information, it may be assumed that formation of the pitcher tube involves the recruitment of a genetic mechanism similar to the one that occur during petal fusion (sympetaly) in *Petunia*, a process known to involve *MAW* and *CHSU* [55]. This assumption stems out from the observation that lateral leaf outgrowth is also severely affected in *maw cshu* mutants displaying extremely narrow leaves [55]. In line with these investigations, similar genetic analysis can be performed and tested, which requires the availability of the genome or transcriptome sequence of *Nepenthes* for gene mining. In recent years, reports on the genome sequences of some carnivorous plants have been made available [161, 162]; these resources may offer additional insights on the evolution of morphological novelties.

## Abbreviations

AN3: Angustifolia3
APUM23: Arabidopsis pumilio23
ARF: Auxin response factor
ARF3: Auxin response factor3
ARF4: Auxin response factor4
ARP: Asymmetric leaves1/roughsheath2/phantastica
ARR: Arabidopsis response regulator
AS1: Asymmetric leaves1
AS2: Asymmetric leaves2
BP: Brevipedicellus
CDKs: Cyclin-dependent kinases
CIN: Cincinnata
CK: Cytokinin
CLV: Clavata
CRC: Crabs claw
CSHU: Choripetala suzanne
CUC2: Cup shaped cotyledon2
CUC3: Cup shaped cotyledon3
CYC1: Cyclin1
DCL4: Dicer-like4
DPA4: Development-related PcG target in the apex4
ETT: Ettin
FIL: Filamentous flower
GA: Gibberellin
ga2ox1: GA2-oxidase1
ga2ox2: GA2-oxidase2
GA20ox: GA20-oxidase
GARP: Glutamic acid-rich protein
GRF5: Growth-regulating factor5
GRN: Gene regulatory network
HD-ZIPIII: Class III homeodomain-leucine zipper
HIRA: Histone regulator A
IAA: Indole-3-acetic acid
ig: Indeterminate gametophyte
INO: Inner no outer
IPT7: Isopentenyl transferase 7
KNOX1: Class-1 knotted-like homeobox
KN1: Knotted1
KNAT1: Knotted-like from arabidopsis thaliana1
KAN: Kanadi
KAN1: Kanadi1
KAN2: Kanadi2
KAN3: Kanadi 3
KRP: Kip related proteins
L1: Layer 1 of shoot apical meristem
*Le*PHAN: *Lycopersicum esculentum* PHANTASTICA
Lgn-R: Liguleless narrow-reference
LOB: Lateral organ boundaries
MAT: Mean annual temperature
MAW: Maewest
mir164A: microRNA164A
miR165: microRNA165
miR166: microRNA166
NAC: NAM No apical meristem
ATAF: Arabidopsis transcription activation factor
CUC: Cup-shaped cotyledon
NPH3: Non-phototropic hypocotyl 3
ns1: Narrow sheath1
ns2: Narrow sheath2
OSHB: *Oryza sativa* homeobox
OSHB1: *Oryza sativa* homeobox1
OSHB3: *Oryza sativa* homeobox3
OSHB4: *Oryza sativa* homeobox4
PALM1: Palmate-like pentafoliata1
PHAN: Phantastica
PHB: Phabulosa
PHV: Phavoluta
PIN: Pin-formed
PIN1: Pin-formed1
PIN2: Pin-formed2
PIN3: Pin-formed3
PIN4: Pin-formed4
PIN7: Pin-formed7
PRC2: Polycomb repressive complex2
PRS: Pressed flower
PUF: Pumilio/fem-3 mRNA binding factor
RDR6: RNA-dependent RNA polymerase6
REV: Revoluta
RS2: Roughsheath2
SAM: Shoot apical meristem
siRNA: Small interfering RNA
STM: Shoot meristemless
TAA1: Tryptophan aminotransferase of arabidopsis 1
TAS3: *Trans*-acting small interfering RNA precursor RNA
tasi-RNA: *Trans*-acting small interfering RNA
TCP: Teosinte-like1: cycloidea and proliferating cell factor1
WOX: Wuschel-related homeobox
WOX1: Wuschel-related homeobox 1
WUS: Wuschel
YAB: Yabby
YAB2: Yabby2
YAB3: Yabby3
YAB5: Yabby5
YUC: Yucca
YUC124: Yucca1/yucca2/yucca4
YUC1246: Yucca1/yucca2/yucca4/yucca6
ZmPIN1: *Zea mays* pin-formed1.
